# Attitudes of Medical Students towards Conflict of Interest: A National Survey in France

**DOI:** 10.1371/journal.pone.0092858

**Published:** 2014-03-26

**Authors:** Bruno Etain, Lydia Guittet, Nicolas Weiss, Vincent Gajdos, Sandrine Katsahian

**Affiliations:** 1 AP-HP, Hôpitaux Universitaires Henri Mondor, Creteil, France; 2 INSERM U1086 Cancers & Preventions, CHU de Caen, Caen, France; 3 AP-HP, Neurological Intensive Care Unit, Neurology Department, Institut Hospitalo-Universitaire-A, Hôpital de la Pitié-Salpêtrière, Paris, France; 4 APHP, Pediatric Department, Hôpital Antoine Béclère, Clamart, France; 5 Université Paris Sud, Kremlin Bicêtre, France; University of British Columbia, Canada

## Abstract

Following recent health scandals in France, the French parliament adopted law n°2011-2012 to regulate ties between physicians and the pharmaceutical industry. The law also requires pharmaceutical companies to publicize financial and other benefits given to medical students. In this context, we administered a survey to medical students in France, in an effort to identify priorities for future education regarding conflicts of interest (COI). This web-based survey encompassed knowledge about, training on, personal exposure to, and opinions on COI among preclinical and clinical students as well as residents. Two thousand one hundred and one (2,101) students participated. Although most students (64.6%) believed that they are able to define what a COI is, they failed to correctly identify several situations as COI (receiving a gift, being offered a meal, being invited to a conference). Most students reported feeling inadequately educated about COI (85.2%). Compared to other class levels, residents were more exposed to pharmaceutical sales representatives. This exposure is highly associated to receipt of gifts (OR 14.51, 95% CI 11.67–18.05). Medical students were aware of potential bias induced by COI with respect to drug prescriptions and research, but felt personally immune towards COI. In our survey, personal research performed by students was more likely to be associated with perception of potential bias on prescription for self (but not for others) than attending a lecture on COI. Promulgating laws that regulate ties between physicians/students and the pharmaceutical industry is a mandatory first step. However, complementary strategies should be implemented within medical schools, in particular, specific training about COI in early medical education.

## Introduction

During their medical school training, medical students at any level are frequently exposed to pharmaceutical marketing and are likely to encounter multiple daily situations that can be considered as potential sources of conflicts of interest (hereafter referred to as “COI”). In 2011, Austad *et al.* reviewed the available literature on this issue mainly among preclinical and clinical students [1]. They concluded that a majority of preclinical and clinical students reported frequent interactions with the pharmaceutical industry. Some situations frequently encountered by students, such as receiving a gift or participating in educational programs sponsored by the industry, are variably perceived as potential conflicts of interests and as having a possible influence on prescriptions. Certain perceptions and attitudes towards the interactions with the pharmaceutical industry appeared to change during medical school when the responses of preclinical versus clinical students were compared. Some medical school-specific COI policies have been implemented in certain countries (mainly in the US and Canada, but also in some European countries) with various levels of tolerance [2], [3].

Following recent health scandals [4] in France, the French parliament adopted law n°2011–2012 on December 29, 2011 to regulate ties between physicians and the pharmaceutical industry. This law requires health experts to disclose all competing interests and establishes a public register maintained by companies that documents all agreements with and payments to anyone involved in health care [5], [6]. This law also includes a section that specifically relates to medical students, stipulating that pharmaceutical companies are required to publicize financial and other benefits given to medical students.

In this context, administering a survey to French medical students is a first step towards collecting qualitative information about knowledge about, training on, personal exposure to, and opinions on COI and could potentially identify priorities for future education regarding COI in France. Prior to administering the survey, we hypothesized that: (1) French medical students poorly identify some frequent situations as potential COI, (2) French medical students perceive their education about COI as insufficient during their training, (3) French medical students misperceive the bias potentially induced by their own COI although perceiving the bias it poses for others.

## Methods

We used a three-step procedure to conduct the survey. The 60-item, web-based survey, which comprised four relevant domains (knowledge about, training on, personal exposure to, and opinions on COI), was developed based on results of a literature review [1], [7]. Two French experts in medical education and conflicts of interest reviewed the list of selected items and asked only for minor revisions. Once the questionnaire was finalized, we chose to include medical students from all class levels in order to test for differences that may have arisen during the course of the curriculum. Indeed, the review by Austad et al. (2011) had indicated certain changes in perceptions and attitudes during the training [1].

We then performed a pilot study by administering the questionnaire to a sample of 20 medical students in order to evaluate their comprehension of the items. At this step, none of the students required any clarification regarding the questions (Step 1).

The survey was then disseminated to an additional sample of 20 medical students to evaluate how thoroughly the items were completed. Because we observed that certain questions had been left blank at this step, we modified the questionnaire to prevent respondents from skipping any questions (Step 2).

In June 2012, we sent an email, followed by two reminders, to the dean of each medical school in France (n = 37 deans contacted), requesting that they send the link to the online survey to all of their students through their intranet/email databases (Step 3). We closed the survey in December 2012 for the extraction of data and the analyses.

Participation was voluntary and anonymous. All medical students were eligible to participate: preclinical (first three years of medical school), clinical (years 4 to 6), and residents (years 7 to 10). Only students who received an e-mail from the dean of their medical school were potential participants; however, the exact number of students contacted by their dean is unknown.

No formal informed consent was required. The local responsible of Commission Nationale Informatiques et Libertés (CNIL) indicated that we were not required to declare the survey to the French authority that oversees the protection of personal data.

All statistical analyses were conducted using the SAS Statistical Package. The mean and standard deviation (SD) were given for the continuous variables. Chi-square tests were used for the comparison of categorical variables. Logistic regression was performed to assess the effects of selected variables on the perceived potential influence of COI on prescriptions (for self and for others). Results were reported using beta, standard deviation, p-values and the odds ratios. P-values<0.05 were considered statistically significant.

## Results

Two thousand one hundred and one (2,101) students from 37 medical schools participated (696 preclinical [PC], 778 clinical [C] and 627 residents [R]; 64% females, mean age 23.1 [SD, 3.1] years). Five hundred thirty-eight (538) students came from the medical schools in or surrounding Paris, 877 from the northern part of France, and 686 from the southern part of France.

Most students believed that they are able to define what a COI is (64.6%), with differences observed across class levels (PC: 59%, C: 70%, R: 64%, P<0.0001). Identification of various at-risk situations for COI differed across class levels (Table 1). Set 1 includes situations that were poorly identified as COI and that included indirect monetary transfers to the student (mainly implicating gifts, meals, training or conferences). Set 2 includes situations that were more successfully identified as COI probably because these imply a direct monetary transfer to the students. With the exception of meal invitations, we observed a moderately increased recognition of COI for all situations as the class level increased. Even though the observed p-values are significant when comparing the responses of students according to class level, the tendency to perceive or misperceive a specific situation as a potential COI remains very similar across class levels. Set 1 of situations remains identified as a potential source of COI by less than 50% of students of any class level, whereas and Set 2 of situations were identified by a majority of students.

**Table 1:** Medical Students' Knowledge of Situations at Risk of COI.

Question or Statement	% of students responding ‘yes’				
	All	Preclinical	Clinical	Residents	P value *
Do you consider the following situation as a COI ?					
Set 1					
Receiving a gift of minor value (book, pen,.) by the PI	27.7	26.5	27.0	36.2	<.0001
Having a close relative employed by the PI	32.9	30.5	37.4	42.3	<.0001
Being invited for lunch/diner by the PI	35.0	41.1	35.9	38.0	.0001
Participating to a training sponsored by the PI	35.6	35.4	40.3	40.8	.0002
Being invited at a conference by the PI	41.5	46.5	41.9	49.4	.0002
Set 2					
Participating to a clinical study paid by PI	56.0	50.1	58.9	73.7	<.0001
Receiving a fellowship by the PI	58.7	55.4	66.7	72.6	<.0001
Being paid as a speaker by the PI	69.2	71.2	78.2	76.3	<.0001
Holding stock shares of a PI	85.5	84.3	92.7	90.7	<.0001
Receiving salary or honoraria by the PI	89.1	85.0	95.1	96.6	<.0001

COI: conflict of interest; PI: pharmaceutical industry.

* P values were obtained from Chi-square tests for homogeneity between preclinical students, clinical students and residents.

Most students reported feeling inadequately educated about COI (85.2%), with few students having attended a lecture (4.3%) or doing any personal research (11.1%) on COI. They expressed interest in knowing their lecturers' COI (66.6%), yet more than half of the students reported that their lecturers do not disclose their COI (66.5%).

Higher-class levels were more exposed to pharmaceutical sales representatives, and this exposure was associated with receipt of gifts (OR 14.51, 95% CI 11.67–18.05) (see Exposure to marketing strategies in Table 2). Table 2 also shows that some opinions differed across class levels, with notable contradictions. Although aware of potential bias induced by COI for others, most students felt immune to personal bias (see Consequences of COI for self and others in Table 2). Many students reported that a COI is likely to occur “from the first euro received” (PC: 41%, C: 48%, R: 58%, P<0.0001). Nevertheless, few (21.4%) felt that attending a meal sponsored by the pharmaceutical industry represented a possible COI. Regarding disclosure of COI to patients or in a public database, fewer residents favored more transparency as compared to preclinical/clinical students.

**Table 2:** Exposure to marketing strategies, potential consequences of COI for self and others, transparency.

Question or Statement	% of students responding ‘yes’				
	All	Preclinical	Clinical	Residents	P value *
Exposure to marketing strategies					
Have you ever met a representative of the PI ?	63.9	18.2	79.4	96.6	<.0001
Have you ever received a gift from the PI ?	62.7	28.1	71.8	89.9	<.0001
Consequences of COI for others					
COI can induce bias in medical training	64.5	65.8	61.8	66.5	.13
COI can induce bias in drugs prescription	87.9	89.3	89.7	84.1	.003
COI can induce bias in research	86.6	82.3	90.1	87.0	<.0001
Self-consequences of COI					
Having received a gift will influence your future prescription	2.4	1.5	2.0	3.7	.27
I consider having a COI when attending a meal sponsored by the PI	21.4	10.8	24.4	29.5	<.0001
Transparency					
Patients should be informed of their physicians' COI	39.4	43.9	40.3	33.3	.002
I favor a public declaration of COI (Ministry of Health website for ex)	65.0	61.9	67.8	64.9	.08

COI: conflict of interest; PI: pharmaceutical industry.

* P values were obtained from Chi-square tests for homogeneity between preclinical students, clinical students and residents.

We performed a logistic regression analysis to test for an association between personal research on and lectures about COI on two items that assessed the perception of potential induced bias in prescriptions for others and for self. Personal research on COI was more likely to be associated with perceived bias for self (but not for others) than attending a lecture on COI (Table 3).

**Table 3:** Association between perceived bias on prescription for others and for self and sources of information on COI.

	Sources of information on COI							
	Personal research about COI *				Attended a lecture about COI *			
	Beta	SD	P value	OR 95%CI	Beta	SD	P value	OR 95%CI
**COI can induce bias in drugs prescription for others** *	0.01	0.11	0.90	1.03 (0.68–1.60)	−0.05	0.16	0.75	0.90 (0.50–1.77)
**Having received a gift will influence your future prescriptions** *	0.50	0.18	0.007	2.69 (1.25–5.44)	−0.04	0.37	0.92	0.92 (0.14–3.18)

COI: conflict of interest; PI: pharmaceutical industry.

* the reference category was the group of students answering ‘no’ to the questions.

Remark : analyses were performed using gender and class level as covariates.

## Comment

Our survey shows a similar picture among medical students as compared to what has been observed in the US, Canada and non-US countries [1], [8]. Indeed, we observed a low rate of proper identification of at-risk situations despite a high exposure to pharmaceutical industry representatives, requests for more education given poor training in faculties, and a perception of immunity to bias despite recognizing that COI may induce bias in others. During their course of medical studies, residents can prescribe medications, but they seem to be insufficiently aware of potential bias that COI may pose with respect to drug prescriptions.

Previous studies [1] regarding perceptions of COI generally included relatively small samples of students (median number: 214; range 17–1523) and were conducted at only one site, mainly in the US and Canada, where policies of regulation have been implemented in past years with various levels of tolerance [2]. Most of them were cross-sectional and included only clinical students. Only a few have included both preclinical and clinical students, thus allowing comparisons of perceptions of COI according to class level. Even if some previous studies were multi-institutional, most of them have not been national [1].

The main potential methodological limitation of this study is the risk of inadequate representativeness due to the participation rate. We were unable to precisely estimate the participation rate for our survey since we did not know how many students actually received an email with the link to our online survey from their dean. We should mention here that this was the first attempt to address such an issue in France, and a certain degree of reluctance from some faculties to disseminate the questionnaire was not definitively excluded. Despite this, we estimate that our survey is likely to provide a representative picture of students' opinions in France. The gender distribution in our study (64% females) is very close to the distribution in the global population (62% according to French official sources). There is an equivalent distribution between class levels (33.1% preclinical, 37.0% clinical and 29.9% residents) and no over-representation of participants from medical schools from a specific region of France (Paris area versus northern or southern parts of France).

This incertitude regarding the participation rate may, at least partly, be counterbalanced by several strengths/advantages: (1) this is the first survey in this domain to be performed in France, thus providing a first overview of an important shortcoming in medical education (2) our sample size is large compared to previous studies (median number of participants around 200) (3) our sample includes not only preclinical and clinical students, but also residents, which provides qualitative data on the potential differences in perceptions according to class level and (4) only few studies have been conducted in Europe, thus not allowing (indirect) comparisons with USA and Canada, where policies have been established.

Promulgating laws that regulate ties between physicians/students and the pharmaceutical industry is a mandatory first step, which France has undertaken through its ‘Sunshine Act.’ This new law should not, however, remain an isolated action, and the results of our survey suggest that complementary strategies should be implemented within faculties. Promoting training about COI in early medical education, defining policies regulating interactions between the pharmaceutical industry and students, or introducing routine pre-lecture disclosures of COI (role modeling) are relevant next steps to be discussed in French medical schools, since such actions stimulate students' critical thinking and could potentially modify knowledge, attitudes and skills. National Medical Student Associations in France could also play a role in the awareness of the industry influence [7], [9]–[12].

In France, due to recent health scandals [4], medical schools and professors are forced to face the population's increased awareness of COI in medicine. They should encourage personal research on COI by students (according to our findings, a strategy that seems relevant to increase perceived bias for self on prescriptions), but also provide pragmatic educational answers (not exclusively in lectures but also in open discussions or brainstorming sessions or any kind of interactive format) about COI to help young future physicians to increase awareness about COI, cultivate strong ethical values and independent evidence-based medical practices.
